# Flexible ureterorenoscopy and laser lithotripsy in children

**DOI:** 10.4103/0971-9261.55154

**Published:** 2009

**Authors:** When-Chan Yeow, Richard Pemberton, Andrew Barker

**Affiliations:** Department of Pediatric Surgery, Princess Margaret Hospital, Roberts Road, Subiaco, Western Australia 6008

**Keywords:** Laser lithotripsy, pediatrics, ureteroscopy, urinary calculi

## Abstract

**Background:**

Flexible ureterorenoscopy (FUR) and laser lithotripsy (LL) are techniques used in the management of upper urinary tract disorders. These techniques, so far established in adults, are now being used in children as well. We report our experience with 26 cases of pediatric upper urinary tract disorders treated using these techniques.

**Methods:**

In the period from 1997 to 2006, FUR was performed in 26 children (14 males and 12 females) in the age group of three months to 15 years with a mean age of 8.2 years. Twenty five were stented prior to undergoing FUR and 24 presented with suspected upper tract stones (17 pelvicalyceal and seven midureteric). Two cases showed JJ stent migration post-pyeloplasty.

**Results:**

Eight cases involved diagnostic procedures. Six excluded the presence of renal calculi, one had focal medullary sponge kidney, and one had calcified papillae. There were 15 cases of therapeutic FUR. Of these, 12 had LL with only one had incomplete stone fragmentation which subsequently passed spontaneously. Other therapeutic procedures included removal of migrated JJ stents and FUR with the basket removal of a midureteric calculus. Three cases failed ureterorenoscopy due to technical difficulties. The overall success rate was 88.5% for FUR.

**Conclusion:**

FUR and LL are valuable minimally invasive techniques for the examination and treatment of pediatric upper urinary tract conditions. Preoperative stenting improves passage of the ureteroscope and with progressive miniaturization of instruments, the lower weight limit will decrease.

## INTRODUCTION

Flexible ureterorenoscopy (FUR) is a common procedure performed on adults for many purposes. Paediatric FUR has become more widely practiced primarily due to the availability of smaller caliber ureteroscopes. Its use in the pediatric population has been most beneficial in urolithiasis, though other upper urinary tract conditions can also be treated.

Many pediatric cases of urinary calculi occur in the aboriginal population in Australia.[1] Cheah *et al.* in their study of 93 western Australian children found 91.4% of calculi were located in the upper urinary tract and only 8.6% were in the bladder.[1] Open nephro-pyelolithotomy or ureterolithotomy, percutaneous nephrolithotomy or extracorporeal shock wave lithotripsy (ESWL) would have been the only options available to treat these upper tract calculi.

FUR and laser lithotripsy (LL) have been demonstrated as safe procedures and to achieve a greater stone-free rate when compared to ESWL, especially with stones greater than one centimeter.[2,3] The minimal invasiveness of FUR, as compared to open surgery, also allows complete assessment of the urinary tract in cases with suspected upper tract stones.

In 2000, van Savage *et al.* reviewed the management of distal ureteric calculi in their pediatric population. They found that calculi 4mm or greater in size are unlikely to pass and thus will most likely require surgical intervention.[4] Our results with pediatric FUR and LL are described.

## MATERIALS AND METHODS

The records of all children who had FUR in western Australia from January 1997 to January 2006 were reviewed. The search found 26 children with 12 females and 14 males. Their ages ranged from three months to 15 years (mean 8.2 years). There were seven aboriginal children.

Ultrasound showed 24 children with urolithiasis, all upper tract calculi (17 pelvicalyceal and seven mid-ureteric). Four children also had intravenous pyelograms (IVP) in their preoperative assessments. They presented with symptoms of pain, hematuria, or had recurrent urinary tract infections. Some were being monitored with serial ultrasounds, which demonstrated persisting and enlarging stone size. Two children underwent FUR to remove JJ stents from the ureter.

Twenty three of the 24 children with upper tract stone disease had retrograde pyelogram and JJ stenting performed prior to FUR. The JJ stent enabled ureteric dilatation for at least two weeks before any attempt was made to pass the ureteroscope. FUR and Holmium: YAG (Lumenis Versa Pulse Powersuite 20W) LL was performed in a manner similar to adults. A 7.5Fr Storz adult flexible cystoscope or a 7.5Fr Olympus flexible ureteroscope were used. A 9.5/11.5Fr or 10/12Fr Cook ureteral access sheath was used in all children who had LL. The ureteroscope was advanced up to the kidney under fluoroscopy over a guide wire and, during LL, a second guide wire was left alongside the ureteroscope for safety. With the ureteroscope in the renal pelvis, each calyx was individually assessed for calculi. Once the calculus was located a 200micron laser fiber was used under direct vision to vaporize the stone to fragments one mm or smaller in size.

Post LL, the children stayed overnight with a 3F ureteric catheter draining the upper tract strapped to a Foley indwelling urinary catheter, which was removed the next day. The cases that were assessed to have edematous ureters post procedure had a JJ stent left in situ which was removed two weeks postoperatively.

Patients were followed up in the outpatient clinic with urinary tract ultrasounds. The average follow-up period was 20 months. The Aboriginal children from the northern regions of western Australia were followed up by their community general practitioners and visiting pediatricians with regular urinary tract ultrasounds.

## RESULTS

**Figure 1:**
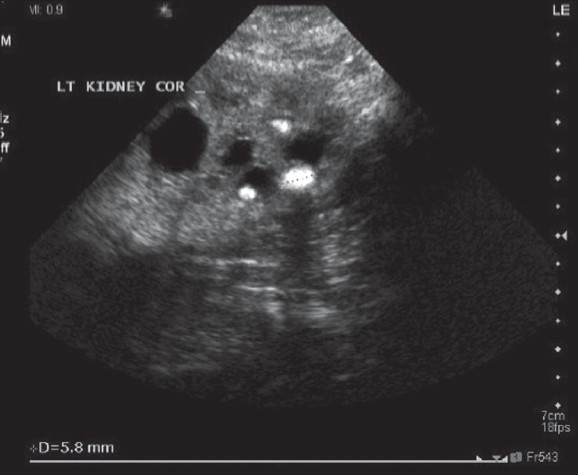
Three-Month-Old Child with Preoperative Renal Calculi on Ultrasound. Ureterorenoscopy Revealed Calcified Papillae

Twenty six patients were treated for upper urinary tract disorders; 24 for presumed urolithiasis and the average stone size was 10.3mm (range three to 21mm). Twenty five children had JJ stenting before FUR. The child not prestented was a three-month-old male who had “open” ureterorenoscopy into his megaureter whilst the bladder was opened for bladder diverticulum repair and ureteric reimplantation. He had suspected urolithiasis on ultrasound. Inspection revealed calcified papillae [Figure 1].

Only eight children underwent FUR [Table 1]. In six, no calculi were seen in the upper renal tract at time of FUR. However, three children had matrix stone seen to wash out after removal of the JJ stent. Calcified papillae and focal medullary sponge kidney were diagnosed in two children. Fifteen children had therapeutic procedures, 13 were for stone disease. One child had a mid ureteric calculus, which on FUR was felt to be small enough to remove with a basket. Twelve underwent LL, with all having successful fragmentation except one, who on ultrasound during follow up still had three to four mm fragments. These were treated conservatively and passed spontaneously.

**Table 1:** Summary of fexible ureterorenoscopy outcomes

	N (subtotal)
**Therapeutic**	**(15)**
Laser lithotripsy:	
Successful fragmentation	11
Incomplete fragmentation	1
Retrieval JJ stent	2
Ureteroscopic basket removal mid ureteric calculus	1
**“Diagnostic”**	**(8)**
Negative for calculi	6
Focal medullary sponge kidney	1
Calcified papillae	1
**Failed (successfully converted to open)**	**(3)**
Ureter not accessible	1
Narrow calyx infundibulum	1
Poor vision	1

Two children had pyeloplasties performed. One child, during removal of the JJ stent had the stent snap within the ureter. The ureteroscope was passed and the proximal part of the stent was located at the level of the pelvic brim and snared with a basket to successfully remove the fragment without complications. The second child who had pyeloplasty had proximal stent migration and so there was no distal end to grasp at cystoscopy. The ureteroscope was passed and the JJ stent was found midureter and removed with a basket without complications.

There were three failed FUR cases. In a 2.5 year old child with a 12 by 21mm right renal calculus the ureter was too edematous for guide wire insertion after her JJ stent was removed at the time when ureteroscopy was attempted. In a 10-year-old male, the ureteroscope was unable to be manipulated successfully due to a narrowed infundibulum of the calyx. The third case was an 11-year-old male who originally had calcium oxalate staghorn and a lower pole calculus. His LL was abandoned due to reduced vision from the large stone burden being treated with lasertripsy. All three children were successfully converted to open pyelonephrolithotomy. The overall success rate was 88.5% (23/26).

## DISCUSSION

In our institution, computed tomography (CT) was not routinely performed for assessing urolithiasis in the time period of this paper because of its radiation-associated cancer risk. Instead, patients first undergo an ultrasound and occasionally an IVP, if required, to confirm the diagnosis. Currently CT is performed more often using lower dose targeted non contrast scanning with helical CT for urolithiasis.

All the eight patients who underwent “diagnostic” FUR were diagnosed as upper tract calculi preoperatively with calculi ranging from three to five mm. The FURs were performed to treat the calculi with LL. The surgeon saw stone fragments when he removed the JJ stent in three children just prior to FUR. In the six children (where no calculi were seen) we believe the calculi were dislodged during their procedures.

In our series, 25 patients had stenting performed prior to any attempts to pass the ureteroscope. We found this beneficial as the kidney was relieved of obstruction, converting an emergency situation to an elective operation. It also allowed passive ureteric dilatation. However, two general anesthetics are required.

During ureteroscopies, the ureteric orifice can be difficult to intubate and traverse, as it is narrow. In Singh *et al*. the tip (first 2cm, approximately 9Fr) of a hydrophillic 14Fr ureteral access sheath is used to dilate the ureteric orifice.[5] Our access sheaths used were smaller. The benefit with access sheath use is that the ureteroscope can be passed multiple times, if required, without causing ureteric trauma especially at the orifice.

In the earlier patients, when experience with FUR in children was less, all patients had post-operative JJ stenting as well. More recently, if there was no significant ureteric edema (assessed at time of operation) and minimal ureteroscopic passages, a ureteric catheter was inserted up to the renal pelvis and strapped to a bladder catheter overnight. This enabled quick, simple removal as a single unit the next morning prior to discharge.

No complications were encountered other than the failed FUR due to technical or anatomical difficulties precluding safe completion of the operation. The major complications reported in the literature include stricture formation, ureteric perforation and ureter avulsion. We have no known long term complications for the current follow-up period.

## CONCLUSION

FUR and LL are valuable minimally invasive techniques for management of pediatric upper urinary tract disorders. Usually only one treatment is required. Calculi can be followed into the renal pelvis if they migrate up the ureter during treatment and hard cysteine stones are also treatable. FUR is also useful in other conditions such as those requiring renal pelvis examination, biopsy and foreign body retrieval. Preoperative stenting improves passage of the ureteroscope and with progressive miniaturization of the instruments; it will be able to be performed in smaller children.

## References

[1] Cheah WK, King PA, Tan HL (1994). A review of pediatric cases of urinary tract calculi. J Pediatr Surg.

[2] Lam JS, Greene TD, Gupta M (2002). Treatment of proximal ureteral calculi: holmium:YAG laser ureterolithotripsy versus extracorporeal shock wave lithotripsy. J Urol.

[3] Wollin TA, Teichman JM, Rogenes VJ, Razvi HA, Denstedt JD, Grasso M (1999). Holmium: YAG lithotripsy in children. J Urol.

[4] Van Savage JG, Palanca LG, Andersen RD, Rao GS, Slaughenhoupt BL (2000). Treatment of distal ureteral stones in children: similarities to the American urological association guidelines in adults. J Urol.

[5] Singh A, Shah G, Young J, Sheridan M, Haas G, Upadhyay J (2006). Ureteral access sheath for the management of pediatric renal and ureteral stones: a single centre experience. J Urol.

